# Nanohybrids as Protein-Polymer Conjugate Multimodal Therapeutics

**DOI:** 10.3389/fmedt.2021.676025

**Published:** 2021-09-08

**Authors:** Pallavi Kiran, Amreen Khan, Suditi Neekhra, Shubham Pallod, Rohit Srivastava

**Affiliations:** ^1^Department of Biosciences and Bioengineering, Indian Institute of Technology Bombay, Mumbai, India; ^2^Center for Research in Nanotechnology and Science, Indian Institute of Technology Bombay, Mumbai, India

**Keywords:** polymers, protein conjugates, nanomedicine, drug delivery, diagnostic

## Abstract

Protein therapeutic formulations are being widely explored as multifunctional nanotherapeutics. Challenges in ensuring susceptibility and efficacy of nanoformulation still prevail owing to various interactions with biological fluids before reaching the target site. Smart polymers with the capability of masking drugs, ease of chemical modification, and multi-stimuli responsiveness can assist controlled delivery. An active moiety like therapeutic protein has started to be known as an important biological formulation with a diverse medicinal prospect. The delivery of proteins and peptides with high target specificity has however been tedious, due to their tendency to aggregate formation in different environmental conditions. Proteins due to high chemical reactivity and poor bioavailability are being researched widely in the field of nanomedicine. Clinically, multiple nano-based formulations have been explored for delivering protein with different carrier systems. A biocompatible and non-toxic polymer-based delivery system serves to tailor the polymer or drug better. Polymers not only aid delivery to the target site but are also responsible for proper stearic orientation of proteins thus protecting them from internal hindrances. Polymers have been shown to conjugate with proteins through covalent linkage rendering stability and enhancing therapeutic efficacy prominently when dealing with the systemic route. Here, we present the recent developments in polymer-protein/drug-linked systems. We aim to address questions by assessing the properties of the conjugate system and optimized delivery approaches. Since thorough characterization is the key aspect for technology to enter into the market, correlating laboratory research with commercially available formulations will also be presented in this review. By examining characteristics including morphology, surface properties, and functionalization, we will expand different hybrid applications from a biomaterial stance applied in *in vivo* complex biological conditions. Further, we explore understanding related to design criteria and strategies for polymer-protein smart nanomedicines with their potential prophylactic theranostic applications. Overall, we intend to highlight protein-drug delivery through multifunctional smart polymers.

## Introduction

Advancements in the development of biomaterials have provided promising alternatives for the conventional modalities of diagnostics and therapeutics. Specifically, the use of biocompatible polymers in localized therapy (1), trigger-based therapy (2), and targeted delivery of drugs (3) open novel avenues for efficient therapeutic systems. The distinct physicochemical properties of nanomaterials enable enhanced retention time in blood, easy surface functionalization allows customized utility in different diseases, and enhanced permeability and retention (EPR) effect causes localization of nanoparticles in cancer (4, 5). Nanomaterials encapsulated inside a specific polymer shell have demonstrated excellent efficiency of targeted payload delivery and increased blood retention (6). The increased blood retention capability can be associated with the increased molecular weight of the protein-polymer conjugate, leading to decreased kidney clearance. Some polymers exhibit a characteristic transition at a molecular level and physical level to triggers such as pH, temperature, and ion concentration (6, 7). The nanoparticles composed of polymers such as chitosan and polyethylene glycol (PEG) offer increased stability over metallic nanoparticles and exhibit excellent biocompatibility (8, 9).

Proteins, on the other hand, perform essential biological functions and their hampered synthesis or dysregulation is responsible for different diseases. Functional proteins such as different kinds of enzymes and clotting factors are routinely used to treat diseases and medical conditions (10). With certain modifications of enhancing stability, many other proteins can also act as potential therapeutic agents. However, free therapeutic protein molecules are very susceptible to degradation because of the changes in pH, temperature, or the concentration of certain ions and proteases. Moreover, they might elicit an immune response, and have a rapid blood clearance rate, which necessitates stabilizing the protein molecules and preventing their degradation (11).

Polymers have been used in theranostics for a long time. Their abilities to form a gel and exhibit sol-gel transition have been exploited in many fields (12, 13). Polymers are often used as carriers in many targeted drug therapies that require shielding from the immune system and enzymatic degradation. Polymer coating decreases the surface energy of the materials by blocking the active/ reactive sites that can interact with other materials or cells. This not only increases the stability of the materials but also helps them escape the immune system (14). Because of these properties, they offer tremendous applications in targeted drug delivery.

A special class of nanomaterials, known as conjugated polymeric nanomaterials (CPNs), has recently gained increased attention for theranostic applications as an alternative to metallic and inorganic nanomaterials. The myriad of theranostic applications of CPNs stem from their π-conjugated backbone facilitating electron exchange and an intrinsic ability to interact with light (3). The diagnostic applications of CPNs are based on multiple techniques such as fluorescence imaging (15), two-photon imaging (16), near-infrared (NIR) imaging (17), photoacoustic imaging (18), and multimodal imaging. Minimally invasive therapeutic applications exploit photothermal therapy (PTT) and photodynamic therapy (PDT). Their candidature as efficient theranostic agents is bolstered by their superior macrophage evasion ability, higher bioavailability, and enhanced solubility over free protein therapeutics after suitable modifications (19, 20). The physicochemical properties of CPNs can be easily tuned by the choice of polymer (21). Protein-polymer conjugates combine the stability of polymeric particles and the therapeutic action of proteins to achieve maximum effect. The conjugation of polymers such as PEG and poly(vinylpyrrolidone) (PVP) with therapeutic protein provides functional and structural advantages. Especially, the biodegradable polymers conjugated to therapeutic protein can encapsulate cytotoxic drugs for targeted delivery to cancer cells benefiting multimodal therapy (22–24). Another strategy to target cancer cells includes combination therapy which involves administration of multiple therapeutics to achieve a similar effect. One of the therapeutics can be a protein molecule involved in pathways that cause cell death. For example, proapoptotic peptide (25) can be used in combination with chemotherapeutic drugs to achieve higher tumor suppression. Protein-polymer conjugates have exhibited superior efficiency in many diseases as compared to free protein therapeutics. The protein therapeutics conjugated with PEG have found applications in acute lymphoblastic leukemia (26), hepatitis C (27), and chronic kidney disease (28). Many protein therapeutics such as enzymes, antibodies, peptides involved in the inflammation cascade, and proteins involved in blood clotting have improved their efficiency after conjugation with PEG.

Different forms of PEG, with modifications which impart desired qualities, are also being investigated with proteins for theranostic applications (29). The first therapeutic application of protein-polymer conjugates that were commercialized was shown in the treatment of severe combined immunodeficiency disease (SCID) which involves deficiency of adenosine deaminase (28). Adagen is a commercial product that was approved by the Food and Drug Administration (FDA) in 1990 for the treatment of SCID. It consists of an enzyme, adenosine deaminase, covalently conjugated to multiple strands of monomethoxypolythelene glycol. Apart from Adagen, other commercially available PEG-protein therapeutic conjugates include SMANCS (for liver and renal cancer), Somavert (for acromegaly), Krystexxa (for chronic gout), and Jivi (for hemophilia A). Apart from these, numerous candidates are in different clinical phases. The range of diseases that can have improved therapy due to the incorporation of PEG illustrates the capability of these peculiar molecules as therapeutic agents. Even though PEG illustrates remarkable efficiency for drug delivery applications, some studies reporting issues of high susceptibility of PEG to bind to serum proteins leading to mononuclear phagocyte clearance system (MPS) clearance (30), complement activation, and generation of anti-PEG antibodies (31) discourage over-reliance on this polymer. Other polymers that are excellent alternatives to PEG include polysarcosine, which is a polypeptoid made up of naturally occurring amino acid—sarcosine (32), synthetic polymers like poly(N-vinylpyrrolidones) (PVP) and polyoxazolines (33), and zwitterionic polymers like poly(carboxybetaine) (34). While polysarcosine offers advantages like excellent water solubility, lower cytotoxicity, decreased immunogenicity, and easy one-pot synthesis, PVP and polyoxalizones offer lower degradation and tunable properties, biocompatibility, and efficient renal clearance, respectively. The advantages of poly(carboxybetaine) include easy synthesis, high stability, highly tunable properties because of the presence of multiple functional groups, excellent anti-fouling properties, and low preparation cost. The availability of these different kinds of PEG alternatives enables development of customizable therapeutics as per the specific requirements in different clinical conditions (33). The conjugates of protein and polymer have a goal of linking the hybrid biomaterials with a potential range from site-specific synthesis to various alternatives. Modifications to protein synthetic peptides could also be described in various applications (35). The difficulty arises when modification involves a single protein in two or more distinct places with different conjugation groups. At times, the choice to do so is limited due to a lack of chemoselectivity and biomolecular targeting groups (36). Here, we have tried to describe various conjugation methods relating protein and polymer properties. Further, we explore various applications and challenges encountered during the conjugation chemistry of polymer-protein conjugate synthesis.

## Polymer Protein Conjugate

### Properties for Designing Polymer-Protein Conjugates

For an effective conjugation, the choice of polymer and compatible protein is important depending on their property, functionality, and application from a myriad of molecules available. Properties like residual activity, aggregation, and solubility highly affect the conjugation degree (37). Also, the requirement of model protein and polymer drawn among commercially available references should be done accordingly.

#### Selection of Polymer

Basic criteria guiding proper polymer identification include molecular weight, degradability, shelf-life, immunogenic reactions, and related toxicities (38). Polymers of small size tend to degrade and clear faster from the body. Also, polymers with low immunogenicity should be considered. The size of the polymer also affects bioactivity as larger polymers many times yield conjugates that have lower bioactivity. Only a few polymers like trehalose and zwitterionic polymers show improved physical (storage) stability whereas stimuli-responsive polymers may assist in environment-responsive protein drug delivery (38, 39). Polymer flexibility relies on length, compatibility with proteins for forming complex structures, and chemical nature affecting the overall architecture of the conjugate (Shu et al., 2013). Another important factor is polymer topology; for example, brush-like, hyperbranched, or dendritic topologies provide good characteristic features compared with their linear counterparts while forming polymer bioconjugates (40–43). Viscosity analysis is also an explicit requirement as the anti-polyelectrolyte effect causing the change in polymer conformation tends to increase the intrinsic viscosity (44, 45). PEG analogs, stimuli-responsive, biomimetic, and degradable polymers represent a group of polymers explored in polymer-protein conjugation. PEG-conjugated therapeutic proteins are widely used, biocompatible, and commercially available with different functional groups, and have FDA approval. PEG conjugation majorly helps the protein to evade the immune system by steric shielding, and increased conjugated protein vicinity helps improve the shelf-life. Considered a “stealth” molecule, PEG is also a hydrophilic, neutral, and non-fouling molecule with low opsonization rates (46). N-(2-hydroxypropyl)methacrylamide (HMPA), another hydrophilic biocompatible polymer, has been used as a reliable conjugate with protein as a drug delivery agent for their unique structural, physicochemical, and biological properties. Likewise, poly(HMPA) randomly coils delivering an acceptable self-assembled conformation in aqueous media, and when attaching a hydrophobic side chain, the copolymer is considered for multi-component conjugations (47).

#### Selection of Protein

Distinctive monomer sequences and accurate 3D structures form high-order superstructures for active functioning (48). The selected protein and its polypeptide backbone should be examined for denaturation and could turn to be challenging at the later stages of purification (49). Protein degradability by protease, biocompatibility, peptide sequence, functionalization in a specific position, and tunability all are important aspects for protein-polymer conjugates (50). Also, the protein's active site must be predefined and the binding motif should be away from the conjugation site (46). Proper assessment of protein aggregation and solubility is essential for therapeutic proteins. Reduced solubility at higher concentrations causes the protein to aggregate and precipitate. This phenomenon highly affects the protein dynamics, structure, and bioactivity which also occur due to altered molecular bonds including hydrogen and electrostatic interactions further causing protein degradation. Since proteins are least soluble at their isoelectric point, any modifications to protein structure can alter the solubility of the protein. Different intrinsic and extrinsic factors influence the solubility of the protein corresponding to the number of charged amino acids and the chemical structure on the protein surface (51). A few amino acids like lysine, tyrosine, disulfide-bonded cysteine as well as the modified C- and N- terminal amines have importance in residue-specific and site-selective conjugation (52–55). While dealing with nucleic acid conjugation with a polymer, flexibility in sequence and protected physiochemical properties similar to non-conjugated counterparts is desirable and has prominence. Stability and scalability can limit the use of oligonucleotides as the processes can directly affect their structural integrity (50).

### Conjugation Methods

**Figure 1:**
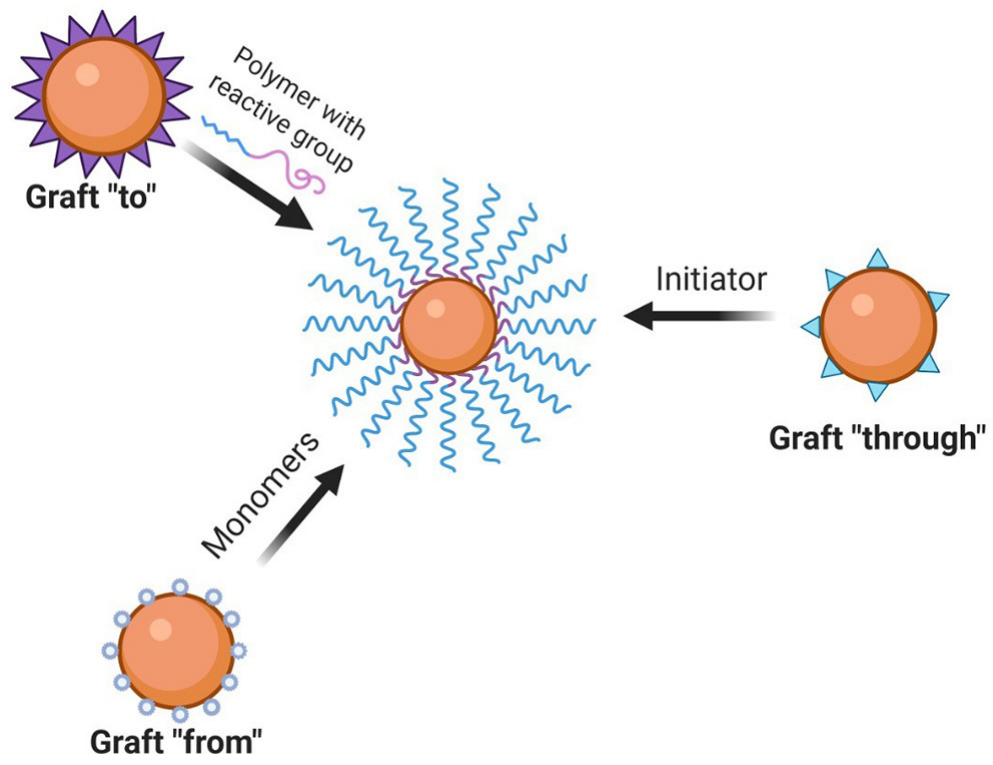
Types of polymer protein conjugation methods (created with biorender).

Proper linkage and chain-growth assessment of the choice of materials are designed to follow the intended activity. For this purpose, conjugation between protein and polymer is employed by three synthetic strategies namely grafting-to, grafting-from, and grafting-through as represented in Figure 1 (50). Out of which, the most extensively exploited are the initial two as grafting-through uses the processing of larger macromolecules. The classification can be postulated depending on the initiator attached to either polymer or peptide and sequential occurrence of the polymerization step.

#### Grafting-To

As a common and relatively simpler approach, grafting-to is used in many conjugation reactions (56–58). In this method; preparation of the backbone and side-chain is done separately (59). Protein-polymer conjugates, due to the polymer having a functional group, are capable of linking to another complementary counter group present on the protein or peptide. For an effective graft-to reaction, parameter control with high coupling efficiency, and the maintenance of the appropriate condition is important for gaining good yield and activity (60). Limited grafting density, an unreacted side chain, associated steric hindrance, difficulty in purification and recognition of the position, and the number of grafted polymers are also encountered in this approach (60). Various modifications have been completed to overcome these drawbacks. For instance, functionalization by Ring-Opening Metathesis Polymerization (ROMP), well-known for its speed, high productivity, low dispersed polymer, and control over polymer properties, has been explored widely in therapeutic and diagnostic applications (61, 62). Due to reported difficulties associated with regulating polymeric weight when polymerized with graft-from ROMP, researchers started to explore graft-to ROMP. One such study suggested lysozyme functionalization with PEGylation of polynorbornenes by graft-to ROMP. Here, a reactive linker, norbornene carboxylic anhydride, was capped on the polymer which assisted attachment of the polymer to the protein's lysine residue. With mildly alkaline conditions, the total conversion of the protein to the polymer-protein conjugate as the biochemical reaction proceeded and retention of native secondary structure was observed. When checked on the scaffold of a Qβ-coated protein self-assembled virus-like nanoparticle substrate, multivalent PNBs showed minimal structure distortion, moreover, toxicity on murine fibroblasts was found to be negligible (63). Reversible addition-fragmentation chain transfer (RAFT) polymerization, in particular, is one of the control radical polymerization techniques offering a straightforward way to synthesis a protein-polymer conjugate utilizing protein reactive groups with a functional chain transfer agent (CTA). This technique provides a wide range of functionalization of polymers for polymer-protein construction (64–67). Vanparijis et al. demonstrated diverse conjugate formation by a graft-to RAFT CTA as different reactive moieties of acrylates and methacrylamides were linked to lysine or cysteine residues as the reaction proceeded toward polymerization. The N-hydroxysuccinimide, pentafluorophenyl ester, maleimide, and pyridyl disulfide moiety presented by the RAFT agent formed conjugates with amino acids. The efficiency of the bioconjugate was examined on bovine serum albumin and avian serum albumin. Among the conjugates, pentafluorophenyl- and pyridyl disulfide-functionalized polymers showed higher efficiency (64).

#### Grafting-From

Addressing issues with improved grafting density and a larger backbone, structures obtained through the grafting-from method are assisted by various super-soft elastomers and stimuli-responsive materials useful in drug delivery formulations (59). Another advantage of the graft-from method is the possibility of conjugate purification from low molecular weight solutes through the simple treatment of dialysis and do not require gel filtration to remove unreacted polymer (68). In this approach, the substrate is first functionalized which acts as an initiator for polymerization (preferably radical) of a monomer. While performing polymerization, the amino acid residues should not have the reactive center to interfere with normal biological activities by undergoing side-chain reactions with undesired groups. On the other hand, the polymerization process should not cause protein denaturation demonstrating a sign of instability and chemical alterations (60). As stated in graft-to, experiments have been performed to prove the effectiveness of graft-from over graft-to. For instance, polymerization of monomethoxy poly(ethylene glycol)-methacrylate from 2-bromoisobutyramide initiators and derivatives of chymotrypsin were conjugated by atom transfer radical polymerization (ATRP) (69). The growth of the polymer on the protein surface, *in situ*, is assisted by ATRP after the initiator gets attached to the protein tuning protein functionality (70). When synthesized with graft-to which is usually considered as a conventional conjugation method, graft-from retained better results with acceptable enzyme activity and desired polydispersity indices. The reaction accompanied functionally distinct and defined polymer-protein conjugates with useful diverse physical and chemical properties of therapeutic importance (69). Another application of chymotrypsin-related ATRP-assisted controlled growth involves stimuli-responsive polymers upon proximity to the vicinity of the initiator on the protein to give protein-polymer conjugates with intact polymer or enzyme activity and stability. This not only allows the change in the stoichiometric ratio between protein and initiator in the protein-initiator complex but also to make improvements in grafting densities. Poly(N-isopropyleneacrylamide), and poly[N,N'- dimethyl(methacryloyloxyethyl)ammonium propane sulfonate] are a few such temperature-responsive polymers (70, 71). A recent similar study was conducted where grating-from RAFT polymerization-conjugated proteins and transiently responsive polymers delivered protein and hydrophobic immunomodulators to dendritic cells. Here, bovine serum albumin protein was made to react with pentafluorophenyl (PFP)-functionalized trithiocarbonate RAFT CTA. The dioxolane moieties in the polymer-protein conjugate allowed a pH-triggered transition. Hydrolysis of hydrophobic dioxolane into diol under acidic conditions caused the dissolution of self-assembled conjugates confirming the structural change (64).

#### Grafting Through

Unlike other methods, grafting-through does not differ much and closely resembles grafting-from. It comprises the polymerization of biomacromolecules as macromonomers and provides the possibility of bioconjugation with every unit of peptide or protein. Many reactive species can be attached to the polymeric backbones forming various structures, and these side chains can be used to act as biologically active moieties. Due to large chains and reactive groups, polymerization may be hindered by the steric effect, which can be controlled by comonomers (59, 60). Polymerization of macromolecules is an important aspect before proceeding with polymer-conjugate formation. Dan et al. synthesized a series of high grafting density molecular brushes of poly(2-oxazoline)s by the grafting-through approach with narrow molecular mass distribution. Oligo- and poly(2-oxazoline) were used as macromonomers for polymerization by aqueous ATRP. Among which, oligo(2-methyl-2-oxazoline)methacrylate showed similar behavior to the commercially available OEGMA475 in terms of divergence from first-order kinetics, low dispersity, and linear molar mass growth as ATRP proceeded (72). The structure is thus reported for the synthesis of complex biomaterials and can further be conjugated with antibodies acting as a drug delivery agent for active targeting (73). Another research group, Thang et al. reported diblock peptide-polymer conjugates synthesis by RAFT polymerization from MARGD, a methacrylamide monomer containing pending RGD peptide with sequences of Phe-Gly-Arg-Gly-Asp-Ser for enhanced cell adhesion. The study delivered a novel and effective strategy for well-defined polymer protein conjugates with a diverse range of peptides and polymers (50, 73). Researchers have also been focused on the elucidation of monomers binding to flat surfaces by combining computation simulation and experiments. Preeta et al. helped in specifically addressing the effect of the special density of the surface-bound monomer-related molecular insight on the formation of attached polymers through the grafting process. For this purpose, the lattice-based Monte Carlo model using the bond fluctuation model scheme and experimental validation utilizing graft-through free radical polymerization was carried out. It was noticed that the surface-bound polymer units boosted the grafting-through process although it prevented the growth of extra free growing chains (74).

Flexibility and multifunctionality demand specificity with improved efficacy to overcome the traditional methods. Apart from the few above-described methods and as an upgrade, precision conjugation can provide a constructive platform to achieve the desired aim. It follows the concept of specific *in situ* growth (SIG) and intrinsically-disordered polypeptide fusion (IPF) for next-generation polymer-protein conjugate formation depending on the nature of the methodology. SIG is a chemical-based method that offers site-specific linkage to the protein of interest followed by *in situ* growth from the protein-initiator conjugate through control polymerization techniques including ATRP, RAFT, and Ring-opening polymerization. The second approach, IPF is a biological method in which intrinsically disordered protein (IDP) is genetically fused to the N- or/and C- terminal of the protein inside the plasmid vector, further expressing the protein and purification to obtain a precisely defined protein IDP conjugate (75). Recently, another approach, different from the current conjugation techniques, has been investigated using RAFT copolymerization. Here, the monomer used contained protein. The acrylate group was attached to the lysine residue of enzyme horseradish peroxidase through a polyethylene glycol (PEG) linker to improve functionalization yield. Predominantly, the HRP-RAFT copolymer synthesized by RAFT copolymerization with N-acrylomorpholine was able to preserve enzyme activity (76). Overall, advancement in protein-polymer conjugations has been dramatically escalated to best fit the field of biomedicine with sustainable biological activities.

### Characterization and Evaluation of Polymer-Protein Conjugates

Although advantageous multi-component characterization is difficult compared to simple molecular therapeutic systems. Protein-polymer conjugation undergoing this magnitude of modifications and stress during in-process conditions becomes prone to thorough analysis and characterization. Ranging from physicochemical characterization which includes solubility, surface group functionalization, and charge density specifically on the surface of the molecule to stability studies and characteristic evaluation that requires a multitude of processes. The techniques can be particular to the type of conjugation and construct (21). Likewise, Majoros et al. utilizing a carrier system poly(amidoamine) (PAMAM) dendrimer synthesized multifunctional cancer therapeutic nanodevices. The modifications to terminal primary amine groups governed the fate of conjugation or specific targeting. Partial acetylation neutralized a few of the amine groups on the dendrimer limiting non-specific interactions during drug delivery. Analytical techniques of potentiometric titration confirmed the number of primary and tertiary amino groups. Fluorescein isothiocyanate (FITC), Folic acid (FA), and methotrexate (MTX) were conjugated to the PAMAM dendrimer by thiourea, amine with ester derivative, and ester bond, respectively. The characterization of this G5-Ac3(77)-FITC-FA-OH-MTXe trifunctional conjugate was done by gel permeation chromatography for molecular weight, nuclear magnetic resonance spectroscopy for functional group estimation, and high-performance liquid chromatography and UV spectroscopy for drug content estimation (78). The techniques of separation, characterization, and evaluation of a conjugate can also be material-dependent. For instance, the PEGylated protein conjugate class, which has importance in modern therapeutics, follows strict clinical efficacy and safety guidelines. Molecular weight, size, and property variation in conjugated to non-conjugated counterparts are too small to be detected and require a series of multiple checks whose characterization and purification techniques are represented in Table 1. Bioassays, pharmacokinetic assessments for evaluation of plasma clearance rate, immunogenicity, and toxicity studies have also been performed for characterization purposes (79, 80).

**Table 1:** Characterization and purification techniques with their physicochemical properties.

**S. No**.	**Physicochemical properties**	**Techniques**
1.	Molecular mass Size Shape	Matrix-assisted laser desorption ionization-time of flight mass spectrometry Size exclusion (gel permeation) chromatography Membranes Capillary electrophoresis Gel electrophoresis Light scattering
2.	Protein folding	Circular dichroism
3.	Electrostatic charge	Cation and anion exchange chromatography Capillary electrophoresis Isoelectric focusing Isoelectric point gel electrophoresis
4.	Relative hydrophobicity	Hydrophobic interaction and reversed-phase chromatography
5.	Antigen-antibody interaction	Enzyme-linked immunosorbent assay Western blotting Surface plasmon resonance
6.	Relative solubility	Aqueous two-phase systems

Through the advances in protein-polymer conjugation, various techniques are also evolving. Biophysics characterization dealing with dynamics of parameters involved in folding has seen a trend with the development of improved conjugates. Differential scanning fluorimetry and intrinsic tryptophan fluorescence techniques have been used by a research group to find thermodynamic parameters of Gibbs free energy, enthalpy, and entropy of folding. The comparison between conjugates, the influence of varying attachment sites, polymer identity, and length facilitated analysis. The investigating group supported both the techniques as replacements of circular dichroism and functional chemical and thermal stability (81). However, with more complex conjugation and synthesis, the validation of various techniques requires the establishment of more profound approaches. Also, imaging moieties, multifunctional nanomedicine, and 3D architecture of conjugates directly affect the linker design when discovering new characterization techniques for better product quality (21, 82).

## Application of Polymer-Protein Conjugation in Drug Delivery

Various protein-polymer conjugation therapeutic modalities are being explored worldwide toward developing multifunctional platforms. Proteins face critical issues when administered to the body like low stability, proteolysis, and fast clearance, thus to overcome these issues and enhance their activity, they are tailored with polymers to provide protection and longer circulation in the bloodstream. The collective demands of the biomedical and health care realm like drug delivery, genetic alterations, protein transportation, hybrid gels, carriers enhancing pharmacokinetic activities, and bioactivity are also being met by incorporating polymers (77, 83). Drug delivery is a prominent application with strategically demanding applications that involve a variety of conjugation methods like reversible deactivation radical polymerization (RDRP) and ROMP for improving efficacy and bioavailability (84). Conjugation not only enhances the stability and solubility but also prevent the phagocytosis of therapeutics. It synergistically produces hybrid materials by combining the properties of individual components and meeting optimal therapeutic needs (85).

### Current Progression

PEG is presently a leading biopolymer for conjugation with proteins. As an FDA-approved biomaterial, PEG has been widely used for decades in the pharmaceutical world and has contributed significantly to various other industrial and biomedical applications (86). With low toxicity in humans, selectivity, specificity, high solvation, and other unique properties, it has been coupled with many proteins. Most of the applications of these hybrid conjugates utilize the benefits of PEGylated proteins to increase the stability and solubility and remain catalytically active inside the body. Enzymatic and selective chemical approaches are the most commonly used approaches for PEGylation (87, 88).

Despite the long list of advantages, PEG has a few drawbacks as well like non-biodegradability, renal clearance due to huge size (large molecular weight), and anti-PEG antibodies leading to hypersensitivity, which have been debatable concerns in recent times (46, 89). Many alternative materials are being worked on in labs in the early stages of development. Dextran, dextrin, polysialic acids, hyaluronic acids, hydroxyethyl-starch, poly(2-ethyl 2-oxazoline), and polypeptides are some of the examples of other polymer materials under experimentation explored in conjugation with proteins (88). With technical advancements in synthesis methodologies, synthetic polypeptides, also known as protein PEGylation, have recently arisen as biomimetic polymers with tunable degradation and responsive features. Self-assembly, modifiable side-chain structures, and additional desired functional groups have increased the scope of biomaterials (35). Numerous PEG-conjugated protein therapeutics have been approved by the FDA and are in clinical use including Pegasys [interferon- α2a (IF-2a)], PegIntron [interferon- α2b (IF-2b)], Neulasta [granulocyte colony-stimulating factor (G-CSF)], Somavert (growth hormone receptor antagonist), Mircera (epoetin-β), and Krystexxa (uricase) (90–93).

Living radical polymerization (LRP) or RDRP is a modern method employed for conjugation of proteins with synthetic polymers in a highly controlled manner. The method improves the homogeneity and efficacy of the conjugate (94). Smart polymers mimic biopolymers by easily undergoing reversible changes and acting as gates or switches that exploit and control the activity of proteins (84). Also, recently developed function-dependent conjugates are being worked upon synergistically to counter drawbacks and meet requirements simultaneously. ATRP and RAFT are the most extensively explored techniques of RDRP methods for these bioconjugation processes for developing “smart stimuli-responsive polymers” (85). Smart polymers are also suitable as PEG alternatives for typical biomedical applications. They respond to a certain external stimulus like pH, light, and temperature undergoing predictable changes which are generally reversible and thus preferable to perform desired roles (86, 94).

**Figure 2:**
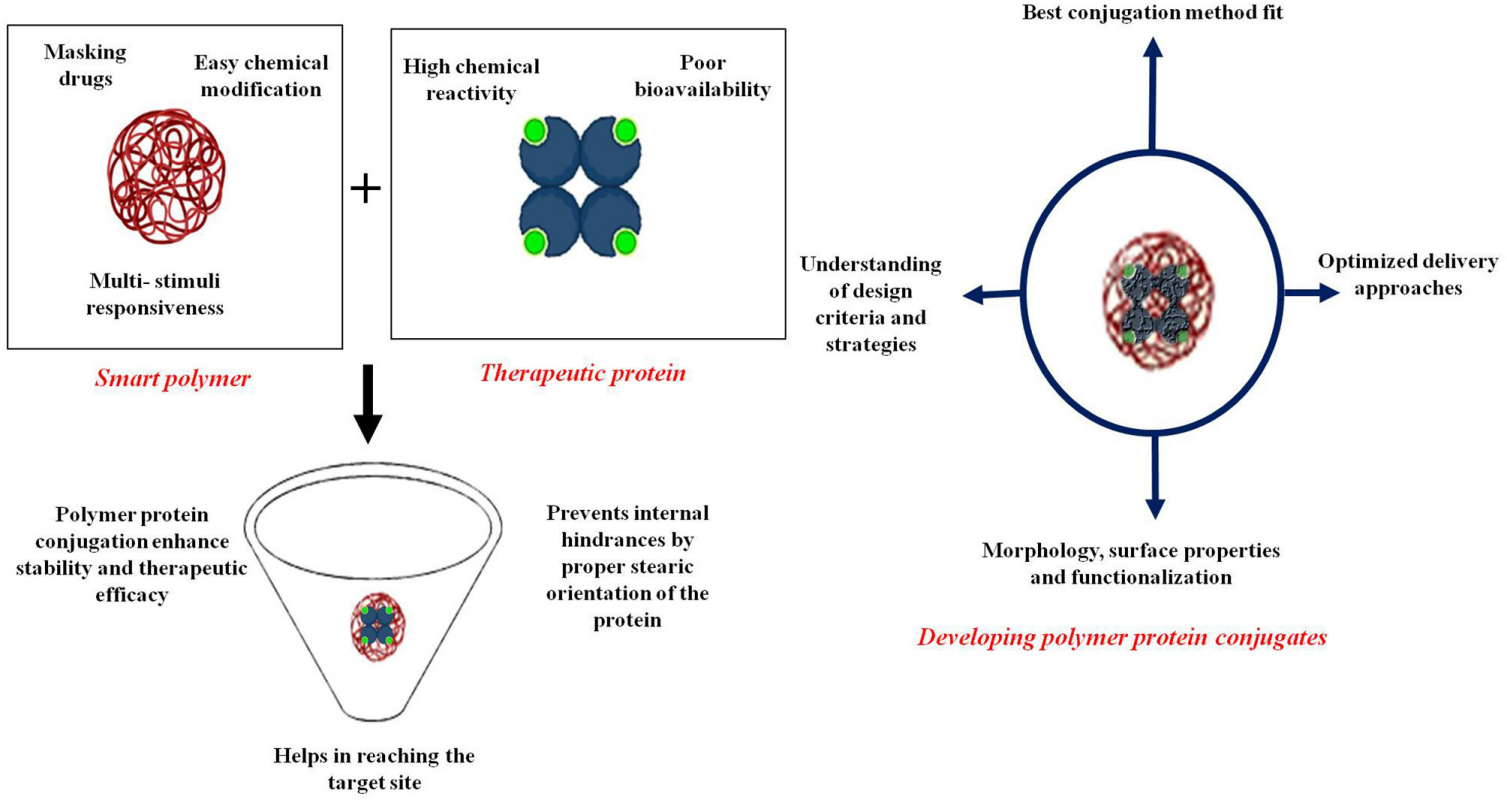
Applications of various protein-polymer conjugates in the wide spectrum of biomedical fields (created with biorender).

Most potential applications of protein-polymer conjugates are oriented towards the pharmaceutical arena as shown in Figure 2, especially by using PEGylated conjugates for drug delivery (95). A significant number of these conjugates have also been found to stay stable in strong organic solvents, retaining the stability required for bioreactor and biosensors-based applications (85). Controlled release of biotin from the streptavidin/poly(N, N-diethylacrylamide) conjugate is another example of stimuli-based smart polymer biohybrid conjugates (77, 96). Temperature and photosensitive polymers are also being conjugated with therapeutic proteins to enhance benefits at the desired sites usually by changing their conformation, shifting hydrophobic moieties (collapsing), and/or expanding (rehydrating) (97). With the change in temperature of the external environment around the conjugate, the interaction of these temperature-sensitive polymers and solvents alters abruptly in correspondence to the upper critical solution temperature (UCST) and lower critical solution temperature (LCST). A good number of these thermosensitive polymers which show LCST behavior close to human body temperature are widely used for drug delivery purposes via “smart” hybrid materials (98).

Polyacrylamides, vinylalkylamides, (meth)acrylates, poloxomers, and oxazolines are a few such examples of polymers belonging to this category (16, 99). Poly(N-vinyl caprolactam) (PNVCL) is an intriguing polymer with compelling properties like solubility in both organic solvents and water, low toxicity, high biocompatibility, and an LCST temperature of 33°C. Owing to these excellent properties, PNVCL has attracted the attention of the scientific community and has become useful for a huge range of biomedical applications (100). DMA-co-4- phenylazophenyl acrylate (DMAA) and DMA-co-N-4-phenylazophenyl acrylamide (DMAAm) are two such stimuli-responsive polymers that expand and collapse while photoswitching, thus providing exciting opportunities in drug delivery, diagnostics (especially microfluidic diagnostic), and bioprocessing (84, 97). Nonetheless, this domain of protein-polymer conjugation remains to be explored for overcoming the general bottleneck including precise conjugation chemistry and loss of biological activity in the processing techniques. Herein, we documented a list of protein-polymers conjugates with their applications for disease treatment, or other related therapeutic purposes, that are under research and also in the early stages of development (Table 2).

**Table 2:** List of protein-polymer conjugates for various biomedical applications.

**S. No**.	**Protein**	**Polymer**	**Method**	**Application**	**Development stage**	**References/clinical trial identification number**
1.	Bovine serum albumin (BSA)	Phenylpiperazine acrylamide monomer	Grafting from and atom transfer radical polymerization (ATRP)	Protein transportation	*in vitro*	(101)
2.	Transportan 10 (TP 10))-based chain transfer agent (TP-CTA)	Poly[oligo(ethylene glycol) methyl ether acrylate]-bpoly(n-butyl acrylate) (TP-POEGA-b-PBA)	Reversible addition-fragmentation chain transfer (RAFT)	Cell penetration and protein transportation	*in vitro*	(102)
3.	Bovine serum albumin (BSA)	Phenylboronic acid functionalized poly(N-isopropylacrylamide) (PNIPAAm)	pH-sensitive borate ester bond	Targeted drug delivery	*in vitro*	(103)
4.	Organophosphate hydrolase (OPH)	Poly(carboxybetaine) (pCB)	Zwitterionic polymer conjugation	Biotherapeutic delivery via non-invasive methods	*in vitro*	(104)
5.	Bovine serum albumin (BSA)	Poly(ε-caprolactone-co-lactide)-b-poly(ethylene glycol)-b-poly(ε-caprolactone-co-lactide) triblock copolymer (PCLA)	Amine group conjugation	DNA delivery based hybrid hydrogels	*in vitro*	(105)
6.	Human serum albumin (HSA)	Hydroxypropyl methacrylate (HPMA)	Self-assembly and *in-situ* atom transfer radical polymerization (ATRP)	Nanovesicles	*in vitro*	(106)
7.	Bovine serum albumin (BSA)	Poly(methyl methacrylate) (PMMA)	Non-covalent bonding	Drug delivery	*in vitro*	(107)
8.	Bovine serum albumin (BSA)	Pyridyl disulfide-functional polymer (2-hydroxy ethyl acrylate)	Disulfide bonds and reversible addition-fragmentation chain-transfer (RAFT)	Controlled release of carbon monoxide	Lab scale development	(108)
9.	Holo-transferrin	Poly(2-vinyl-4,4-dimethylazlactone) (PVDMA)	Azlactone functional bonding and reversible addition-fragmentation chain-transfer (RAFT)	Targeted drug delivery	*in vitro*	(109)
10.	PEGylated lysozyme	Poly(2-(methylsulfinyl)ethyl acrylate) (MSEA)	Grafting and reversible addition-fragmentation chain-transfer (RAFT)	Drug delivery with improved pharmacokinetic activity	*in vitro*	(110)
11.	Silk sericin (SS)	Polylactide (PLA)	*bis*-aryl hydrazone linker	Drug delivery	*in vitro*	(111)
12.	Lysozyme	Pentafluorophenyl (PFP)-ester-functionalized phosphorylcholine (PC) polymer	Ester bond conjugation and reversible addition-fragmentation chain-transfer (RAFT)	Retaining enzymatic and pharmacokinetic activity	*in vivo (preliminary stage)*	(112)
13.	Pyrophosphatase (PPase conjugated to Maleimido-functionalized cyclodextrin).	Poly(N-isopropyl acrylamide) (Ada-PNIPAAm) or poly(oligo(ethylene glycol) methyl ether acrylate) (Ada-POEGMA)	Host-guest interaction	Site-specific mutagenesis	Lab scale development	(113)
14.	Bovine serum albumin (BSA)	Hydrophobic maleimide-functionalized poly(ε-caprolactone) (PCL)	Maleimide-sulfhydryl coupling reaction.	Drug delivery	*in vitro*	(114)
15.	Chymotrypsin modified 2-bromoisobutyramide	Monomethoxy poly(ethylene glycol)-methacrylate	Protein-display initiators and atom transfer radical polymerization (ATRP)	Protein-polymer conjugates	Lab scale development	(115)
16.	Bovine serum albumin (BSA)	Fluorescent PEG-b-poly(N-(2-hydroxypropyl) methacrylamide) (PEG-b-PHPMA)	Ugi reaction	Protein therapeutics	Lab scale development	(89)
17.	Streptavidin	Poly(*N,N-*diethylacrylamide)	Biocomplexation	Biosensors and diagnostics technologies	Lab scale development	(96)
18.	Osteoprotegerin	Poly(ethylene glycol) methyl ether methacrylate (PEGMA) and N-(2-hydroxypropyl) methacrylamide (HPMA)	Reversible addition-fragmentation chain-transfer (RAFT) polymerization	Bone loss disorders	*in-vivo*	(116)
19.	Bovine serum albumin (BSA) with a maleimide-functionalized chain transfer agent (CTA)	N-isopropylacrylamide (NIPAM)	Reversible addition-fragmentation chain-transfer (RAFT) polymerization	Drug delivery	Lab scale development	(117)
20.	Horseradish peroxidase (HRP) enzyme	*N*-acryloylmorpholine (NAM) via polyethylene glycol (PEG) linker	Reversible addition-fragmentation chain-transfer (RAFT) polymerization	Industrial and biomedical purpose	Lab scale development	(76)
21.	Avidin	Poly(carboxybetaine methacrylate) (pCBMA) polymers	Atom transfer radical polymerization (ATRP)	Preparing bioconjugates	Lab scale development	(118)
22.	Myoglobin	Poly(ethyl ethylene phosphate) (PEEP)	Bioconjugation	Protein-polymer conjugates	Lab scale development	(119)
23.	Dipeptide (Boc-Cys-Trp-OMe)	Poly(1-vinylimidazole) (PVim) chains	Grafting-from protocol based on thiol-mediated radical polymerization	Bioimaging	*in vitro*	(120)
24.	Superfolder green fluorescent protein (sfGFP) with 2-pyridyl potassium acyltrifluoroborate (KAT)-hydroxylamine amide	Polyethylene glycol (PEG) chains	Bioconjugation	Protein PEGylation	Lab scale development	(121)
25.	Microbial transglutaminase (MTG) with Z-QG synthetic short peptide	Polyacrylamide	Site-specific conjugation	Immunological biosensing	Lab scale development	(122)
26.	Bovine serum albumin (BSA)	poly(methyl methacrylate) (PMMA)	Self-assembly	Drug delivery	Lab scale development	(123)
27.	Lysozyme	Poly(*N*-acryloylmorpholine) (PNAM) and poly(oligo ethylene glycol methyl ether methacrylate) (POEGMA)	Reversible addition-fragmentation chain-transfer (RAFT) polymerization	PEG alternative development for protein conjugation	Lab scale development	(37)
28.	Transferrin	2-(2-Methoxyethoxy) ethyl methacrylate (MEO2MA)	C- reactive protein-based conjugation	Therapeutics	*in vitro*	(124)
29.	Chromoprotein neocarzinostatin	polystyrene-maleimide	Bioconjugation	Cancer therapeutics	*in vivo*	(125)
30.	Chymotrypsin	Poly(sulfobetaine methacrylamide)-block-poly(N-isopropylacrylamide) (CT-pSBAm-block-pNIPAm)	Atom transfer radical polymerization (ATRP)	Enhanced pH and temperature stability	Lab scale development	(101)
31.	Human fibroblast growth factor 21	Polyethylene glycol (PEG)	–	Non-alcoholic steatohepatitis	Phase 2 clinical trial	NCT02413372
32.	Interferon beta-1a	Polyethylene glycol (PEG)	–	Relapsing multiple sclerosis	Phase 3 clinical trial	NCT00906399
33.	Recombinant human hyaluronidase	Polyethylene glycol (PEG)	–	Pancreatic ductal carcinoma	Phase 3	NCT02715804
34.	Arginine deiminase	Polyethylene glycol (PEG)	–	Hepatocellular carcinoma	Phase 3	NCT01287585
35.	Proline-interferon alpha-2b	Polyethylene glycol (PEG)	–	Polycythemia vera	Phase 2	NCT03003325

### Other Applications

**Figure 3:**
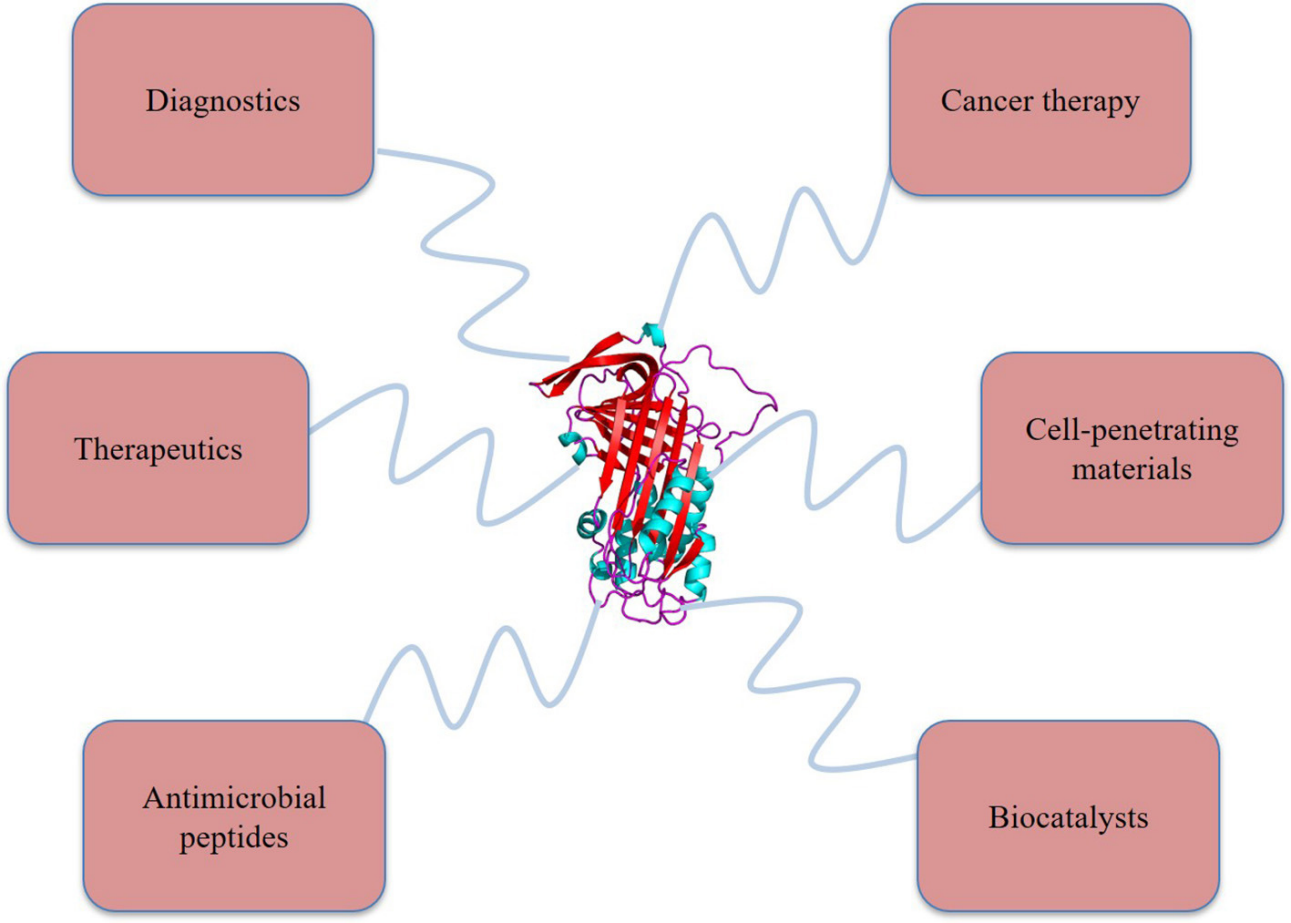
Application of various protein polymer conjugate in the wide spectrum of biomedical fields.

Apart from highly advancing fields such as drug delivery and tissue engineering, CPNs have also established their efficiency as antimicrobial agents. Typically, an antimicrobial peptide (AMP), which is readily degraded by the proteases in free form, is conjugated with a polymer for targeting to the bacterial biofilm. Xie et al. targeted such AMPs conjugated with methacrylate to the biofilm found in dental caries and achieved complete lysis at concentrations as low as 10 μg/ml (126). Ortiz-Gómez et al. developed AMPs conjugated with PEG that prevented biofouling by killing *E. coli* and *P. aeruginosa* from surfaces (127). The ability of materials to cross the plasma membrane of the target cells is crucial in the majority of drug delivery approaches. Certain peptides such as KAFAK exhibit a cell-penetration ability with an anti-inflammatory response which can be used in drug delivery applications. Conjugation with polymers provides proteins with stability to perform their functions. As demonstrated by Chen et al., the cell-penetration ability of a peptide sequence was retained after linking it with a specific polymer (102). CPNs have been related to the field of biocatalysis as well as shown in Figure 3. Because of the enhanced solubility and stability of the protein in non-native environments, protein-polymer conjugates have demonstrated a remarkable ability to catalyze organic aqueous reactions at an organic liquid-water interface. The catalytic activity of a fragile enzyme, benzaldehyde lyase, that is involved in Pickering interfacial catalysis was found to be restored upon conjugation with the polymer, poly(N-isopropylacrylamide) (128). With diverse applications, FDA-approved PEG-protein conjugates and PEG-aptamer conjugates have found their ground as therapeutics (129). As replacements of native enzyme deficiency with increasing red or white blood cell count and neutralization of overactive cytokines or related receptors, conjugates are used widely (130). Hepatitis or Crohn's disease treatment is another application of protein-polymer conjugates. Also being employed to stimulate white blood cells after chemotherapy, conjugates have proven to be an important class of biologics (46, 131). Further, the application of protein-polymer conjugates is increasing in gene delivery and tissue engineering (77).

## Challenges in the Development of Conjugates

**Figure 4:**
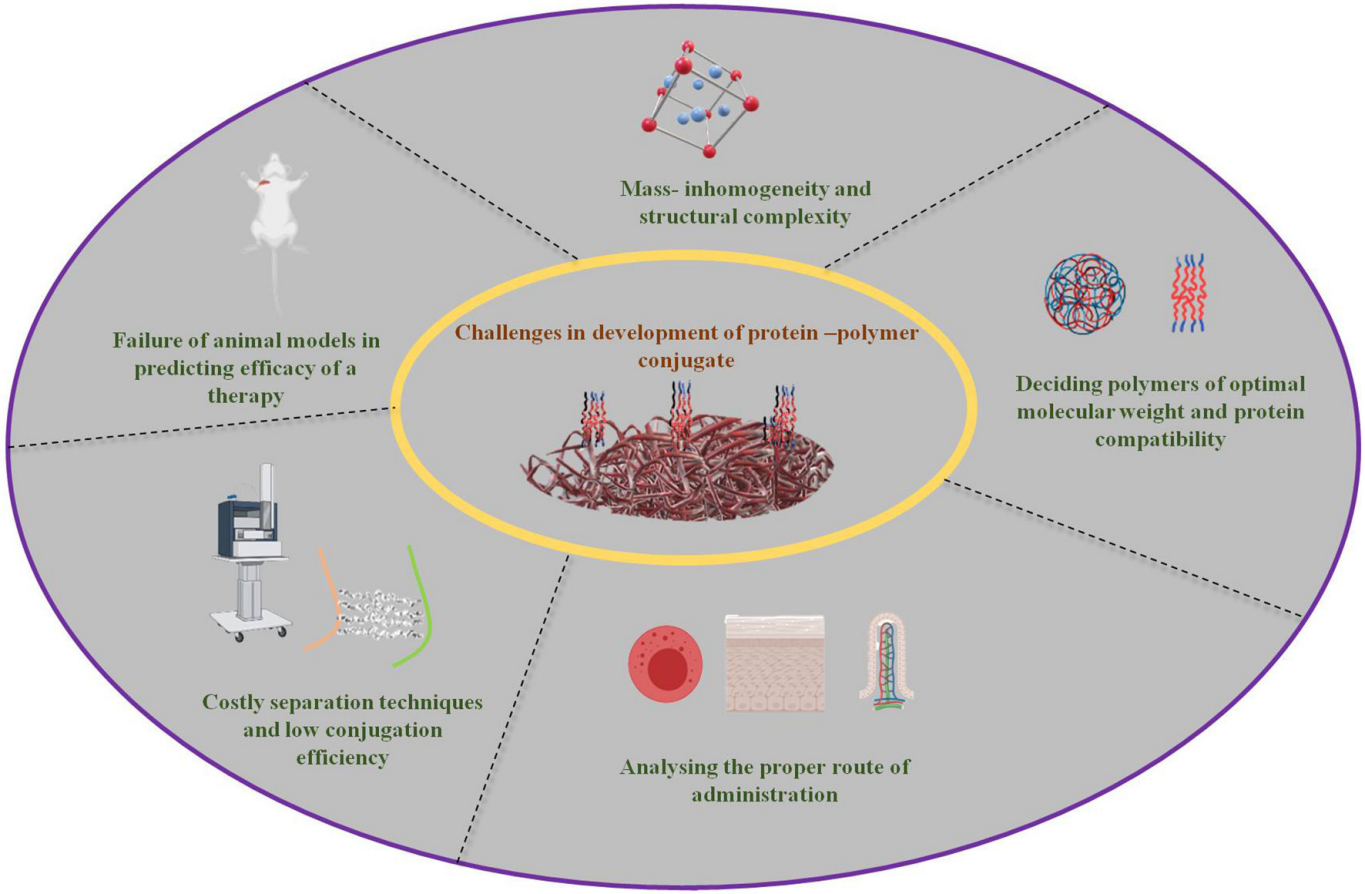
Major challenges for the conjugation of protein and polymers hampering their further developments (created with biorender).

With the recent progression in conjugation methodologies and their extensive development, many more are planned for polymer-protein conjugation for drug delivery and related biomedical applications. Nonetheless, some challenges remain to be confronted despite the technological advancements. A brief overview has been shown in Figure 4.

Structural complexity and unpredictability of the conjugation process of mass inhomogeneity have made simulation-based analysis very complex and challenging, and at times comprehensive practical correlation has become a mandate to overcome. Multiple parameters are involved in the design and extensively affect the properties of the conjugate. The final *in vivo* behavior of the conjugate and its complex physicochemical properties determine its fate and biological efficacy (132). Homogeneity and stability of the protein constructs along with their key linkages also require improvement to enhance the conjugate stability against protease degradation *in vivo* (106). Polymer length, physicochemical affinities, and stoichiometry are equally important aspects when undertaking polymerization reactions and protein conjugation (133).

The size of the protein-polymer conjugate is another crucial factor that determines the fate of conjugates in the bloodstream during circulation, pointing out the importance of choosing an appropriate polymer for the protein. High molecular weight polymers are employed to increase the molecular weight of low weight therapeutic proteins to increase their circulation time in the bloodstream and restore their activity, but too high a molecular weight can easily lead to tissue cytotoxicity that is probably due to the accumulation of the conjugate at non-desired or non-targeted tissues in close correlation with the EPR effect. Thus, optimizing close to the required molecular weight and honing the high selectivity of the conjugates are big challenges yet to be faced. Apart from this, zeta potential or the net surface charge of the conjugate has a prominent effect in deciding the circulation life-time and preventing the aggregation of conjugates. It is preferred that conjugates have a neutral or very low negative charge to prevent opsonization, therefore it is important to choose the start materials by maintaining the balance between the requirement and the zeta potential of the final conjugate. Close supervision in conformational changes should be analyzed and thus toned to maintain stable structures with optimal zeta potentials (133). The appropriate conjugation process, the reaction chemistry of the reacting groups of both the polymer and protein to be conjugated, can be optimized. Grafting-to and grafting-from approaches are routinely used for polymerization, but are dependent on the reactive groups on both the protein and polymer. Properly selecting the protein and polymer can reduce the associated complexities to largely extend the chance of achieving enhanced release at the target site (38).

Non-specificity in permeation is a major challenge that can result in potential toxicity due to the accumulation of conjugates at undesired or off-target locations in chronic administration (110–112, 134). The administration route should be holistically analyzed and approached sequentially in dealing with the absorption barriers first, followed by circulatory, tissue-level, and cellular-level barriers (133). The loss of bioactivity and heavy denaturing of proteins during conjugation processes and *in vivo* circulation are important factors to be considered while designing the protocol. The yield of the polymer polymerization process which is further proceeded by conjugation and drug loading is usually considered to be very low (35, 134). Also, the generation of potential immunogenic agents and their effects after administration cannot be predicted easily. Several concerns have been raised recently regarding the immunogenicity, modifications, and limitations with PEGylated nanomaterials (135). One major frequently occurring issue in the process of PEGylation is the loss of protein bioactivity, which requires attention to release protein moieties similar to the release of drugs. In-depth analysis of pharmacokinetic and pharmacodynamic behavior of therapeutic proteins can be easily employed to optimize the controlled release of protein moieties from the polymer (136). This course of development of bioconjugates from PEGylation to de-PEGylation should be analytically reviewed in close association with the target protein and its characteristics (106, 137).

An elaborate analysis should be performed to confirm that the material is biocompatible. Cytotoxicity, hemocompatibility, carcinogenicity, genotoxicity, and reproductive toxicity are a few such mandatory tests for *in vitro* assessment (138). Polymer toxicity should be necessarily evaluated before further moving ahead in the development pipeline to avoid raising concern for their usage after huge investment (38). Reactive oxygen species (ROS) generation should also be kept under consideration while selecting the initial carriers and designing a proforma (137). The *in vivo* stability, circulation, molecular level-controlled organization, degradation, renal clearance, therapeutic efficacy, half-life in the body, bioavailability, metabolism, off-target intracellular uptake, and accumulation are a few factors that need a detailed systematic examination to analyze the subsequent fate of protein –polymer conjugate systems (139, 140). Thus, it is crucial to establish the physicochemical and biochemical features with optimal density and surface composition of designed conjugate models (102). The conjugates are usually studied and developed under a set of optimized conditions, while in the body, a collaborative presence and highly varying distribution of proteins, hormones, ions, and pH demand well-designed therapeutic nanomaterials. Additionally, intriguing correlation and dependence of *in vivo* estimation should be carefully decoded.

Another set of problems is raised at the translational level of experimentation. Low conjugation efficiency and costly synthetic methods heavily limit the practical usage of many potential protein-polymer conjugates (134). The separation of polymeric monomers, proteins, and protein-polymer conjugates is a very complicated and expensive process (133, 141). Currently in use conjugation experimentation is ideal at the lab scale whereas the process becomes tedious when it comes to mass scale, thus the conjugate needs to be robust, reproducible, and industrially scalable to avoid premature failure in the early stages of development (133, 142). Systematic and thorough optimization of parameters, high-throughput screening of accumulated data, and then building the design approach are important challenges during the developmental process of protein therapeutics (91, 142). Also, all the experiments are firstly performed on a small-sized animal model usually showing promising results, but vary drastically when performed on higher animal models or humans. This heavily delays the developmental process and limits their clinical successes (Shu et al., 2013) (133). Quantifiable changes in the biodistribution and pharmacokinetic aspects of the design and correlating outcomes of computational and theoretical modeling approaches should be critically analyzed (142). There is a significant requirement of efforts to push translational results that are presently limited to animal tests, and their clinical success is still limited (91, 142).

Although the total number of products presently available in the market today still only amounts to a handful and concluding from former paragraphs, many challenges discussed are yet to arise. Future advancements of protein-polymer conjugates will proceed toward interdependent output, correlating physicochemical parameters and biological analysis that will lead to the design of strategies of conjugates with designated and desired characteristics. Hence, while heading toward conjugation-based planning, strategic designs based on predictive modeling and theoretically well-correlated data with a multidisciplinary approach can pave an easier way for translational therapeutics. Cost-effective processes for conjugation have to be explored introducing more affordable solutions, especially for the necessitous population.

## Current Developments and Future Prospects in Protein-Polymer Conjugates

Functionalities of protein-polymer conjugates largely depend on the type and properties of the conjugated polymers. The majority of developments in protein-polymer conjugates are fixated on exploiting and modifying the properties of the polymers. Molecular weight, functional groups, and chain length of polymers affect the properties of the protein-polymer conjugates. There are ample opportunities for improvement in synthesis, purification methods, choice of polymers, and choice of approach. The current trends also predict that future efforts will be focused in the same direction.

### Reducing Toxicity

The pharmacodynamics and pharmacokinetics of CPNs depend largely on the properties of the core material, capping agents, and properties of the polymer used for conjugation. Biocompatibility as calculated in terms of cell viability using a biochemical assay called MTT governs the translation of newly developed materials into preclinical and clinical stages. Some polymers like polyurethanes or capping agents such as N-cetyltrimethylammonium bromide (CTAB) used in the synthesis of anisotropic gold nanostructures are toxic in nature and need to be washed before their use in biological systems. The FDA has approved PEG conjugated with therapeutic protein for commercialization. Due to extensive characterization and a good safety profile, PEG has been most widely used as a conjugating polymer for protein-polymer conjugates (46). PEG imparts excellent pharmacokinetics and biocompatibility to engineered protein-polymer conjugates, but issues of hypersensitivity and antibody formation by the host after repeated exposure persist. These drawbacks demand the development of alternatives for polymers to be conjugated to proteins. Poly(N-(2-hydroxypropyl)methacrylamide) (p(HPMA)) is a well-researched biocompatible polymer which can be copolymerized with other monomers containing pendant drug-reactive groups such as carboxylic acid or ester. It has shown excellent stability against heat and autolysis (47).

### Biodegradability

Materials with biodegradable properties offer a significant advantage over non-biodegradable materials. Safely metabolizing through the excretion system, such materials prevent immune activation. Biodegradable nanomaterials have demonstrated their potential in localized and gradual delivery of therapeutics. The coating of biodegradable polymers over nanoparticles or their conjugation to protein molecules enhances their excretion from the body. Broadly, polymers can be classified into synthetic and natural polymers. Natural polymers include chitosan, hyaluronic acid, gelatin, agarose, and collagen, all of which are biodegradable. Biodegradable synthetic polymers include polylactic acid, polyglycolic acid, polyphosphate, and polysebacic acid, while non-biodegradable synthetic polymers include materials like polymethacrylate, siloxanes, and poloxamers (143). Modifications in the PEG main chain through the introduction of a chemically reactive group can impart biodegradability to the material. The introduction of disulfide groups or acid-labile acetals in the main chain makes the material susceptible to proteolysis or hydrolysis. Apart from PEG, other natural and synthetic polymers are also being investigated for the conjugation of therapeutic proteins. Recently, Boonpavanitchakul et al. synthesized nanosized protein-polymer conjugates containing silk sericin conjugated with polylactide for the development of a pH-responsive drug release system for targeting tumor cells. Natural polymers and their analogs such as hydroxyethyl starch (HES) (47) and other polypeptides, are other examples of biodegradable polymers that have been investigated.

### Stimuli-Responsive

Protein-polymer conjugates have diverse applications in the field of theranostics. Trigger-responsive or stimuli-responsive nanomaterials enable localized delivery and reduce the dosage of the drug. Such ‘smart’ materials can function upon pH-based, ion/cofactor binding-based, temperature-based, or enzyme-based triggers. Polymers such as poly(N-isopropyl acrylamide) (p-NIPAAm) exploit the lower critical solution temperature (LCST) below which it solidifies, and others like poly(acrylic acid) and poly((N, N-dimethylamino)ethyl methacrylate) (p(DMAEMA)) make use of the structural and chemical changes caused due to pH variation. The protein-polymer conjugates of such polymers have been developed for various theranostic applications (85, 144).

## Conclusion

Polymer-protein and drug conjugates have enabled micro- to nanoscale structures with advanced and tunable properties. Designing a system with varied conjugations and compositions enables the improvisation of “grafting-to,” “grafting-from,” and even “grafting-through” techniques. Various types of polymer-protein and drug-conjugated nanoformulations have been described in this review that exhibit therapeutic effects with high drug-delivering efficacy. This efficacy is dependent on multiple factors resulting in numerous challenges as precision increases. Further, we have tried to detail future studies and experimentations, especially involving safety. Assessment with consideration of the long-term effects of increased protein concentration and protein degradation is of critical importance. Even after high-impact technological advancements, a universally acceptable system for the critical assessment of nanosystems is difficult to create, and more complicated structures and conjugation products pose additional challenges. To develop safer protein therapeutics, we have tried to quantify their approaches based on a thorough and meticulous impression of biological processes. The fact that material properties and the selection made on the medicinal requirement of the nanosystem tend to be carried through to the intact conjugated structure has also gained importance. Possibly in a coming trend, we will see multiple proteins and drug encapsulation into polymeric conjugates as potential carriers and drug delivery systems.

## Author Contributions

PK and AK: conceptualization of manuscript and reviewing. PK, AK, SN, and SP: initial drafting and final draft preparation. All authors contributed to the article and approved the submitted version.

## Conflict of Interest

The authors declare that the research was conducted in the absence of any commercial or financial relationships that could be construed as a potential conflict of interest.

## Publisher's Note

All claims expressed in this article are solely those of the authors and do not necessarily represent those of their affiliated organizations, or those of the publisher, the editors and the reviewers. Any product that may be evaluated in this article, or claim that may be made by its manufacturer, is not guaranteed or endorsed by the publisher.
